# Retroperitoneal vascular malformation mimicking incarcerated inguinal hernia

**DOI:** 10.4103/0974-2700.76823

**Published:** 2011

**Authors:** Indu Bhushan Dubey, Anuj Sharma, Ajay Kumar Singh, Debajyoti Mohanty

**Affiliations:** Department of Surgery, University College of Medical Sciences and G.T.B. Hospital, Delhi, India

**Keywords:** Adult, incarceration, inguinal hernia, venous malformation

## Abstract

A 30-year-old man presented to the Department of Surgery with a painful groin swelling on right side. Exploration revealed a reddish-blue hemangiomatous mass in the scrotum extending through inguinal canal into the retroperitoneum. On further dissection swelling was found to be originating from right external iliac vein. The swelling was excised after ligating all vascular connections. The histopathological examination of excised mass confirmed the diagnosis of venous variety of vascular malformation. This is the first reported case of vascular malformation arising from retroperitoneum and extending into inguinoscrotal region, presenting as incarcerated inguinal hernia.

## INTRODUCTION

Vascular malformations are rare diseases resulting from errors of vascular morphogenesis. Malformations of venous origin are usually asymptomatic. Their location into the retroperitoneum is extremely rare. Only very few cases of retroperitoneal vascular malformations are available in literature. We report a case of unusually located venous malformation arising from right external iliac vein presenting as an incarcerated inguinal hernia, managed successfully by surgical excision.

## CASE REPORT

A 30-year-old man presented to surgical emergency with a painful irreducible swelling in the right inguinoscrotal region and high-grade fever for 1 day. He also gave a history of reducible swelling at similar location for last 10 years. There were no features suggestive of intestinal obstruction.

**Figure 1:**
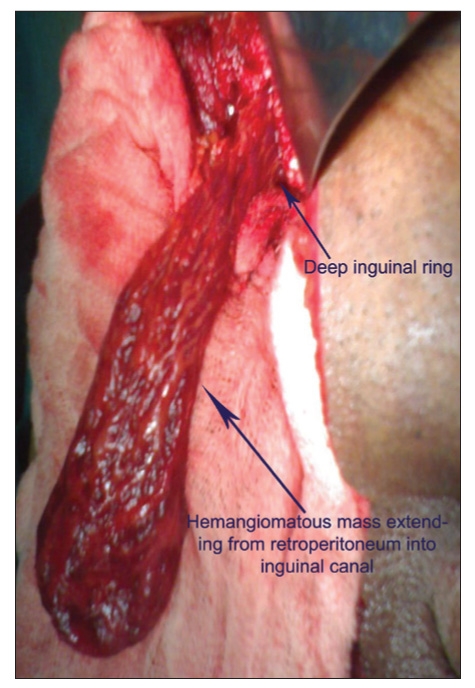
Intraoperative photograph showing hemangiomatous mass, extending from retroperitoneum into inguinoscrotal region

On examination, the patient was febrile. There was a tense, tender and irreducible swelling in right inguinoscrotal region with warm, shiny and erythematous overlying skin. Urgent inguinoscrotal exploration was done with a provisional diagnosis of right-sided strangulated inguinal hernia. A reddish-blue hemangiomatous mass [Figure 1] was found in the scrotum. The mass was extending further into the retroperitoneum through inguinal canal. Further retroperitoneal dissection revealed its origin from right external iliac vein. Cord structures were normal and there was no persistence of processus vaginalis.

**Figure 2a and b:**
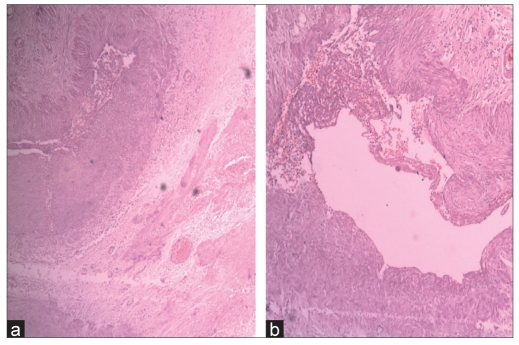
H and E, 40×, Photomicrograph obtained at histological examination showing disorganized muscular wall of veins blending with surrounding soft tissues

The mass was excised after ligating all its vascular connections. Histopathological examination of the excised mass confirmed the mass to be a venous hemangioma (venous malformation). Sections showed disorganized venous walls merging with surrounding soft tissue with areas of focal dystrophic calcification [Figures 2a and b]. Computed tomography of abdomen showed no evidence of other associated vascular malformations. Patient was discharged on fourth postoperative day. No relapse had occurred 12 months after the initial presentation.

## DISCUSSION

Vascular malformations are benign vascular tumor-like growths that usually present themselves at birth or soon thereafter. They do not pose any health problems but occasionally can interfere with various functions of the body depending on their location inside the body. The abnormal dense collections of blood vessels may occur in the muscles, internal organs and mucous membranes or more commonly on skin surface. These lesions are usually classified into capillary, venous, arterial, lymphatic and combined type. Majority of venous malformations are not treated when they are first observed because more than 90% disappear spontaneously by the time the patient reaches puberty.

Retroperitoneal venous malformation is a very rare condition resulting from malformation of angioblastic tissues of fetal life.[1] Only three cases have been reported in the English literature so far.[2] Accurate pre- and intra-operative diagnosis of retroperitoneal venous malformation is difficult and usually delayed because of its location, inaccessible to routine clinical examination. The condition is usually detected only when the clinical symptoms caused by the pressure of the surrounding tissues occur. Presence of capillary variety of vascular malformation in inguinal location in a premature infant presenting as strangulated hernia has once been reported earlier.[3] Retroperitoneal venous malformation extending into the inguinoscrotal region and presenting as strangulated hernia in an adult patient has not been previously reported.

## CONCLUSION

Retroperitoneal venous malformation extending into inguinoscrotal region and presenting as strangulated hernia in an adult patient is an unknown entity. If the patient would have obtained medical consultation for reducible inguinal swelling earlier, an ultrasound Color Doppler examination could have revealed the exact nature of the swelling. The report describes successful management of this rare vascular malformation.
